# Impact of psychosocial, behavioral and lifestyle factors on subjective cognitive complaints and perceived quality of life in a large cohort of Italian breast cancer patients

**DOI:** 10.3389/fpsyg.2022.1015573

**Published:** 2022-11-09

**Authors:** Thomas West, Corrado Cavallero, Rita Ceccherini, Silva Foladore, Daniele Generali, Francesco Versace, Bruna Scaggiante

**Affiliations:** ^1^Lega Italiana per la Lotta contro i Tumori, Trieste, Italy; ^2^Department of Life Sciences, University of Trieste, Trieste, Italy; ^3^Breast Cancer Unit, ASUGI, Trieste, Italy; ^4^Department of Medical, Surgery and Health Sciences, University of Trieste, Trieste, Italy; ^5^Breast Cancer Unit, ASST Cremona, Italy; ^6^The University of Texas MD Anderson Cancer Center, Houston, TX, United States

**Keywords:** cancer-related cognitive impairment, breast cancer, subjective cognitive complaints, adjuvant therapies, chemotherapy, cognitive reserve, sleep quality

## Abstract

The impact of psychosocial and behavioral factors on Cancer Related Cognitive Impairment manifestations is still under debate. Study’s purpose is to determine the prevalence rate of cancer related cognitive impairment in a cohort of Italian breast cancer patients and to evaluate the implication of specific behavioral factors. For these purposes, a total of 233 women (106 breast cancer patients and 127 age-matched controls without oncological diagnosis) completed a questionnaire investigating cognitive functionality (FACT-Cog v3.0), sociodemographic characteristics, clinical information, psychosocial and behavioral factors (cognitive reserve, sleep quality, dietary habits, physical activity). The results indicated a higher prevalence rate of subjective cognitive complaints in breast cancer patients (37%) compared to a representative sample of women in the same age group without an oncological diagnosis (*p* < 0.001). Moreover, breast cancer patients showed significantly lower levels of cognitive reserve (*p* < 0.05) and worse sleep quality (*p* < 0.01) compared to age-matched controls. Further analysis revealed that breast cancer patients reporting subjective cognitive complaints differed significantly from breast cancer patients without subjective cognitive complaints on measures of perceived cognitive abilities (*p* < 0.001) and on the impact of cognitive difficulties on perceived quality of life (*p* < 0.01). Future studies are needed to examine behavioral directed interventions to prevent subjective cognitive deficits in breast cancer patients.

## Introduction

In recent years advances in cancer management strategies have significantly improved survival rates. The reduction in death rates due to the employment of adjuvant therapies has shifted the focus to long-term adverse effects of cancer and its systemic treatments (Mandelblatt et al., 2016) and their impact on the quality of life of cancer survivors.

One of the most common side effects is cancer-related cognitive impairment (CRCI), a term used to describe a plethora of cognitive difficulties regarding memory, attention, language, and executive functions, that affect a large proportion of noncentral nervous system cancer patients (non-CNS), with severe implications on their reintegration into the workforce (Boykoff et al., 2009; Yarker et al., 2010; Duijts et al., 2017), driving abilities (Myers et al., 2015), and adherence on treatment plans (Zogg et al., 2012; Von Ah et al., 2013; Alatawi et al., 2021). Therefore, the goal of addressing CRCI is to help patients cope with their current levels of cognitive function and re-engage and increase their participation in valued roles and activities at work, at school, and in the community. To date, the majority of CRCI research in patients with non-CNS cancer has involved women with breast cancer (BC), who represents approximately 43% of women cancer survivors in Italy (Tagliabue et al., 2021). However, despite the vast literature regarding CRCI in BC patients, the prevalence estimates of subjective cognitive complaints (SCC) vary considerably between studies, ranging from 12% up to 86% (Nelson and Suls, 2013; Janelsins et al., 2017; Whittaker et al., 2022). This lack of consensus regarding SCC cognitive decline in BC patients arises primarily due to methodological issues. First, self-report measures are often non-standardized and lack cut-off values with the result that SCC is not consistently addressed between studies. Secondly, only a small number of studies investigating SCC in women with breast-cancer, focus on psychosocial and behavioral factors that have an impact on perceived cognitive impairment (Myers et al., 2010, 2015) aside from mood disturbances (such as anxiety and depression). Furthermore, most CRCI studies in oncological patients focus primary on its pathophysiological mechanisms or on discrepancies between objective and subjective cognitive measures, but most recent evidence is highlighting the multifactorial nature of CRCI phenomena, and the strict correlation between psychosocial, lifestyle, and cognitive variables on its manifestations (Ahles et al., 2012). But, despite the significant repercussions of CRCI on social functioning in oncological patients, only a few studies have investigated the role of behavioral and lifestyle factors and their role on perceived cognitive functioning (Henneghan, 2016).

The purpose of the present study was to determine the prevalence rate of SCC in a cohort of Italian women with BC by using the cut-off values provided for the FACT-COG questionnaire – Version 3.0 (Dyk et al., 2020), and to explore many potential factors associated with perceived cognitive impairment. The potential factors of interest included age, education level, current occupation, cognitive reserve, sleep quality, dietary habits, level of physical exercise, presence of comorbidities, stage of the disease, and other variables regarding the administration of adjuvant therapies (type of therapy, time since start, time since end). Secondarily, the present study aims to determine which behavioral and lifestyle factors allow to distinguish between BC patients and age-matched controls, and between BC that report CRCI and BC without cognitive complaints, and their impact on perceived cognitive abilities and quality of life, to determine possible non-pharmacological treatments specifically targeted to modifiable behavioral factors.

## Materials and methods

### Study design and participants

A total of 233 participants took part in the study and completed an anonymous electronic or hard copy questionnaire (identical forms) investigating CRCI and other potential behavioral, psychosocial, and lifestyle factors associated with perceived cognitive impairment. A series of 106 BC patients were recruited through clinical oncology practices between Trieste – ASU GI and Cremona – ASST Breast Cancer Units, and 127 age-matched controls enrolled through LILT (Lega Italiana per la Lotta contro i Tumori) – Trieste (Italy). The study inclusion criteria were being female, aged between 25 and 75, absence of known psychiatric or neurological diseases, able to complete a study questionnaire unaided, all stages of BC, and previous and current adjuvant therapies (chemotherapy, radiation, biological, and/or hormonal therapy). Exclusion criteria included previous oncological diagnosis, central nervous system metastases, mental illness, and presence of neurodegenerative diseases.

All the study phases were approved by the University of Trieste Ethical Committee n.106 13.07.2020. All participants provided written informed consent.

### Measures

All participants were given the option to complete an electronic or hard copy anonymous questionnaire (identical forms) comprised of demographic and oncological disease information, the Functional Assessment of Cancer Therapy for Cognition (FACT-Cog) Version 3, and a series of questions regarding intellectual, leisure and social activities across the lifespan, dietary habits, level of physical activity and sleep quality.

*Functional assessment of Cancer Therapy for Cognition (FACT-Cog) Version 3*: The FACT-Cog questionnaire is a specific measure designed to assess cognitive difficulties in cancer survivors, and it is regularly employed in observational and treatment studies (Bray et al., 2017; Janelsins et al., 2017). Version 3 of the questionnaire is a self-assessment scale composed of 37 items investigating perceived cognitive impairment (PCI), perceived cognitive abilities (PCA), comments of others (OTH), and impact of cognition on perceived quality of life (QoL). Numerous analysis (Cheung et al., 2013; Park et al., 2015; Costa et al., 2018; Hajj et al., 2020) confirmed the traditional four-factor structure (impairment, abilities, noticeability and quality of life) for the FACT-Cog v3 questionnaire in cancer patients. Further research (Costa et al., 2018), conducted on healthy adults, concluded that FACT-Cog may perform differently in non-cancer population. The cognitive abilities and QoL factors were generally robust across sample and analytic methods, while older adults’ sample was best described using both broad impairment/abilities factors and specific cognitive domains. Since use of more specific cognitive domains in non-clinical populations might be a reliable procedure only when subjects have a specific knowledge of cognitive functioning, for our study we decided to use the classical four-factor structure of FACT-Cog questionnaire for both groups (BC patients and age-matched controls).

*Sociodemographic characteristics and clinical information*: Demographic information included age, years of education, work status (ranging from 0 = no working to 5 = executive), fluently spoken languages. All patients reported the presence of clinical pathologies. BC participants also reported number, type, and duration of adjuvant therapy, including date of start and completion (used to calculate time since adjuvant therapy) and history of surgical procedure.

*Subjective cognitive complaints*: All four scales were scored from FACT-Cog, including Perceived Cognitive Impairments (PCI-18 items; 0 = never, 4 = several times a day), Perceived Cognitive Abilities (PCA-9 items; 0 = not at all, 4 = very much), Comments from Others (OTH-4 items; 0 = never, 4 = several times a day), and Impact on Quality of Life (QOL-4 items; 0 = not at all, 4 = very much). Higher scores on the FACT-Cog equate to higher perceived cognitive function.

*Psychosocial variables*: A measure of cognitive reserve – CR (ranging from 0 to 34) was obtained through participants’ self-report frequency of intellectual, leisure, and social activities during the lifespan (0 = never, 1 = during childhood adolescence, 2 = during adulthood, 3 = in the last year), and level of social interactions during childhood/adolescence, adulthood and last year (0 = none, 3 = many friends ≥5 and close relationships with some of them) in addition to specific demographic information: education level (ranging from 0 = primary degree to 5 = postgraduate degree, master’s degree, doctorate), work status and number of fluently spoken languages.

*Behavioral variables*: A Dietary Habits Index-DHI (ranging from 0 to 9) was obtained *via* specific scales regarding the frequency of a balanced diet (0 = never, 4 = almost every day), excessive intake of specific food categories (0 = every food category, 4 = none), number of meals a day (0 = more/less than 4 meals a day, 1 = 4 meals a day). A Physical Activity Index-PAI (ranging from 0 to 8) included questions regarding the frequency of aerobic and anaerobic physical activity (0 = less than a time per week, 4 = 5 or more times per week). A Sleep Quality Index - SQI was scored *via* administration of specific questions regarding the level of rest after night sleep (0 = none, 4 = almost every day) hours of sleep per night (0 = less than five, 3 = eight hours or more), sleep quality overall (0 = fragmented/with numerous nighttime awakenings, 1 = continuous/without nighttime awakenings).

Higher scores on the psychosocial scales equate to better levels of cognitive reserve, dietary habits, physical activities, and sleep quality.

To proceed with statistical analysis participants were classified into two groups based on presence or absence of BC diagnosis (BC-patients, age-matched control group) and two more sub-groups based on cut-off scores provided by Dyk et al. (2020) for the FACT-Cog PCI-18 items scale (SCC=PCI-18 ≤ 54, No-SCC=PCI-18 > 54).

### Data analysis

The differences between the number of BC subjects and the age-matched control group reporting SCC were analyzed by Chi-square test. In addition, due to the nature of the variable (presence vs. absence of SCC based on cut-off values reported in “Materials and methods” section), a binary logistic regression analysis was performed to determine the likelihood (Odds Ratios – ORs) of experiencing SCC between the two groups, with 95% confidence intervals (CI), using group (BC vs. age-matched control) as a predictor. The normality of the distribution of variables obtained from the questionnaire was investigated by the Shapiro–Wilk test (*p* > 0.05). Based on the distribution of the variables, *T*-test and Mann Whitney U-Test analyses were carried out to assess possible significant differences in the two groups regarding demographic, cognitive efficiency, and lifestyle variables. Three 2×2 factorial between subjects’ ANOVAs were used to investigate further differences between groups on measures of FACT-Cog questionnaire. Between subjects’ variables were group membership (BC and Age-matched controls), and presence of subjective cognitive complaints – SCC (SCC=PCI-18 ≤ 54, No-SCC=PCI-18 > 54). Post-hoc analysis was performed through Bonferroni correction. Finally, a correlation analysis using Pearson-r correlation coefficient between variables considered in the present study was performed. Moreover, to address the effect of statistically significant correlated behavioral and psychosocial variables on sub-scales of the FACT-Cog questionnaire (PCI-18, PCA, OTH, QoL), linear regressions and multiple linear regression analysis, using the “stepwise” method were used. For the Stepwise multiple linear regression, at each step, variables were chosen based on value of *p*s < 0.05, and the value of *p* threshold of 0.1 was used to set a limit on the total number of variables included in the final model.

The statistical analyses were carried out using the Jamovi analysis software^32^. All statistical tests with *p* < 0.05 are reported as statistically significant.

## Results

### Sample characteristics

Table 1 contains demographical information regarding the study sample. BC group consisted of 106 female participants aged between 32 and 74 years (Mean age = 53.69, SD = 8.78), with an average education level of 13 years (high school degree), and 58% of the participants were currently working at the time of self-report administration. Regarding clinical history, 39% of BC participants reported at least one clinically relevant comorbidity (e.g., diabetes, cardiovascular or rheumatic diseases) and all the participants underwent at least one treatment cycle (or mixed combination) of adjuvant therapies (chemo-, radio-, biological and/or hormonal therapy), the vast majority (91%) had surgery for the oncological disease. As regards to the age-matched control group, 127 participated in the present study. All participants were female, aged between 26 to 75 years (Mean age = 52.46, SD = 11.96), with higher average education (Mean education = 15.90, DS =3.93), and a higher percentage of currently working participants (64%) compared to BC group. Furthermore, when comparing clinical history, the age-matched control sample also reported non-oncological clinical pathologies with a slightly lower incidence (33%) in comparison to BC patients.

**Table 1:** Descriptive statistics for breast cancer (BC) patients and age-matched controls.

	All women (*N* = 233)	BC patients (*N* = 106)	Age-matched controls (*N* = 127)	Value of *p*
Age (M + SD)	53.02 (10.63)	53.69 (8.78)	52.46 (11.96)	0.687
Years of education (M + SD)	15.02 (4.13)	13.96 (4.13)	15.90 (3.93)	<0.001
Comorbidities				0.373
Yes (%)	92 (39%)	41(39%)	42 (33%)	
No (%)	144 (61%)	65 (61%)	85 (67%)	
Currently working				0.409
Yes (%)	93 (39%)	62 (58%)	81 (64%)	
No (%)	143 (61%)	44 (42%)	46 (36%)	
Subjective cognitive complaints (SCC)				<0.001
Yes (%)	60 (26%)	39 (37%)	21 (17%)	
No (%)	173 (74%)	67 (63%)	106 (83%)	
Number of adjuvant therapies				
1 (%)		34 (32%)	–	
2 (%)		39 (37%)	–	
3 or more (%)		33 (31%)	–	
Status of adjuvant treatment				
Ongoing (%)		47 (44%)	–	
Ended (%)		59 (56%)	–	
Type of therapy still ongoing				
Chemotherapy (alone or + other*)		37 (79%)	–	
Other*		10 (21%)	–	
Time from start of adjuvant treatment				
≤1 year (%)		30 (28%)	–	
>1 year (%)		76 (72%)	–	
Time from conclusion of adjuvant treatment				
≤1 year (%)		12 (11%)	–	
>1 year (%)		30 (28%)	–	
>3 years (%)		19 (17%)	–	
Type of adjuvant treatment				
Chemotherapy (alone or + other)		80 (75%)	–	
Other*		27 (25%)	–	

M, mean; SD, standard deviation. *Other types of therapies: radiation therapy, hormonal therapy, biological therapy, or combination.

### BC patients show higher rates of SCC compared to an age-matched control group

**Figure 1:**
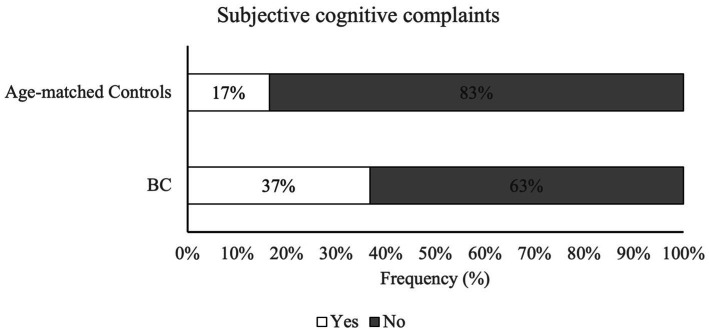
Percentage of subjective cognitive complaints in BC patients and age-matched control group. Patients with BC reported significantly higher rates of perceived cognitive difficulties when compared to age-matched controls. Subjective cognitive complaints were assessed *via* FACTCog – PCI18 subscale and cut-off values provided by Dyk et al. (2020).

Analysis results showed a significant difference in the incidence rate of SCC between the two groups, with a higher percentage in the BC group compared to an age-matched control group [*X*^2^(1) =11.46, *p* < 0.001]. Figure 1 indicates that 37% of BC participants reported a significant level of cognitive complaints that exceeded FACTCog-PCI18 cut-off values, while only 17% of age-matched controls reported comparable severity of cognitive deficits in everyday life. Furthermore, the results from a binary logistic regression analysis reported a significant association between group and SCC [*X*^2^ (1) = 12.78, *p* < 0.001]. The prediction model showed a probability of 2.98 times higher of experiencing SCC in the BC group compared to the age-matched control group (Table 2).

**Table 2:** Binomial logistic regression model for subjective cognitive complaints (SCC).

Model coefficients – subjective cognitive complaints							
						95% confidence interval	
Predictor	Estimate	SE	*Z*	*p*	Odds ratio	Lower	Upper
Intercept	−1.62	0.24	−6.78	< 0.001	0.20	0.12	0.32
Group							
BC – Age-matched	1.09	0.31	3.49	< 0.001	2.98	1.62	5.51

Estimates represent the log odds of “Subjective cognitive complaints = SCC” vs. “Subjective cognitive complaints = No SCC”. SE, Standard Error; Z, z-score; p, *p*-value.

### BC patients show lower levels of cognitive reserve and sleep quality compared to age-matched controls

An analysis of behavioral, lifestyle, and psychosocial variables showed a significant difference for two of the factors considered in the study. The cognitive reserve (CR) of BC subjects (Mdn = 21.00), was lower compared to the control group (Mdn = 23.00). The Mann–Whitney U-Test indicates a statistically significant difference between the two groups of subjects [*U* = 5483.00, *p* < 0.05]. Similarly, sleep quality (SQI) of BC subjects (Mdn = 4.00) was lower compared to an age-matched control group (Mdn = 5.00), and statistical analysis showed significant differences between the two groups [*U* = 5305.50, *p* < 0.01]. Moreover, the analyses showed a near-to-significant difference between the two groups regarding dietary habits [*U* = 5693.50, *p* = 0.052]. BC subjects tend to follow a moderately healthier diet (Mdn = 7.00) compared to age-matched subjects (Mdn = 6.00). Lastly, no significant difference was observed in the level of physical activity (PHI) between the two groups [*U* = 6107.00, *p* = 0.45].

### BC patients referring SCC report lower perceived cognitive abilities and quality of life

**Figure 2:**
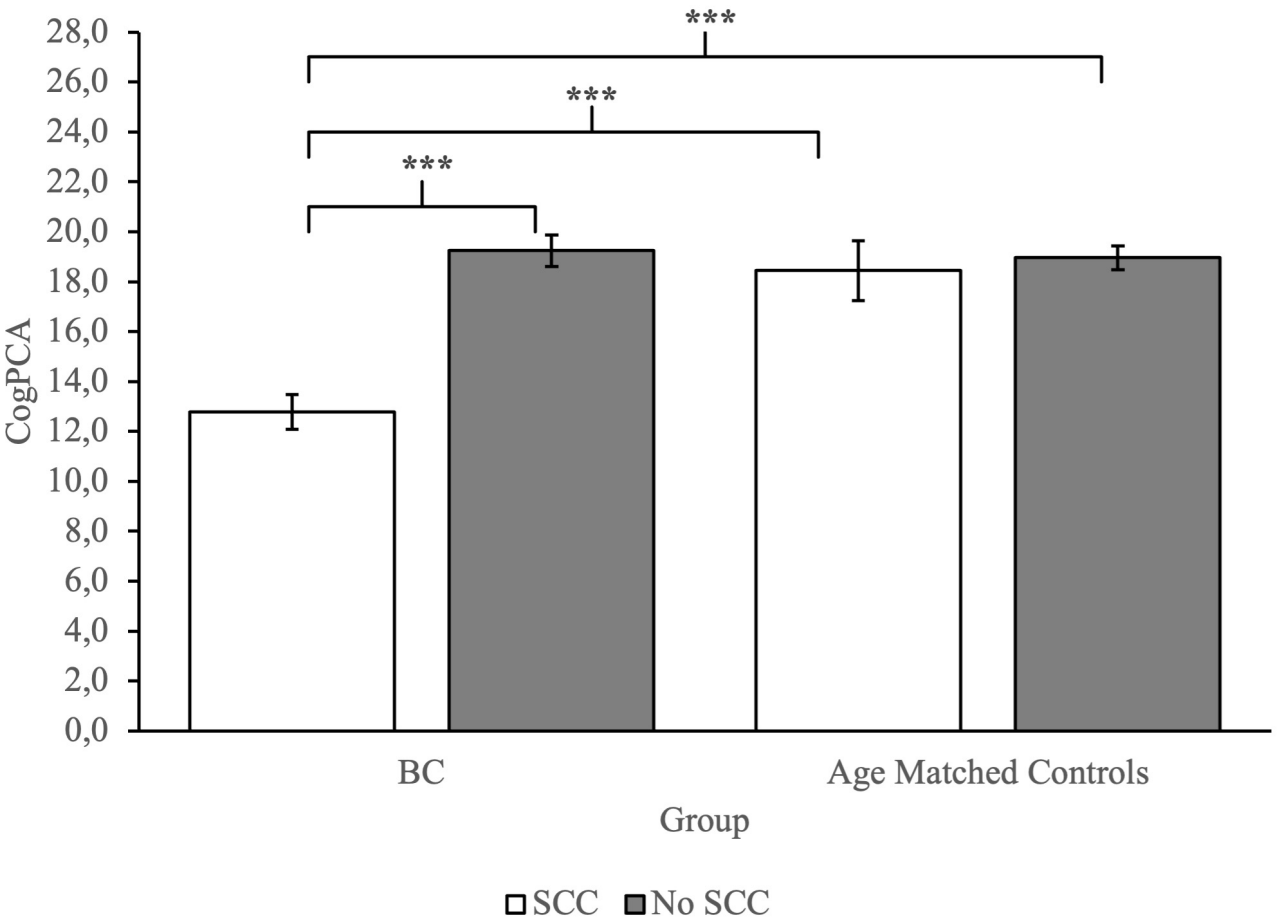
Group comparison between FACTCog Perceived Cognitive Abilities (PCA) measures: BC patients reporting SCC describe themselves as less cognitive skilled compared to BC patients not referring SCC and age-matched controls. ***p < 0.001.

**Figure 3:**
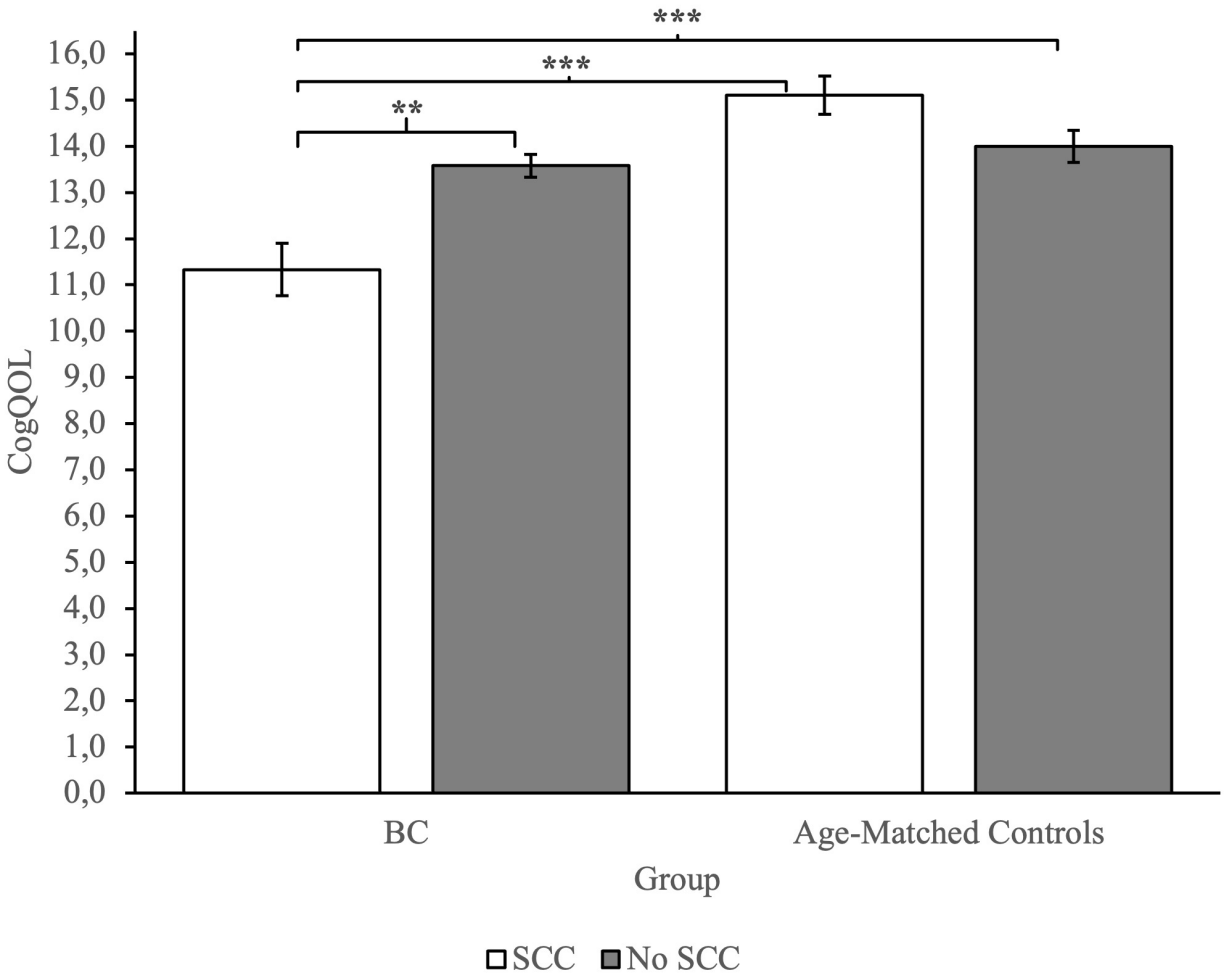
Group comparison between FACTCog Impact of perceived cognitive impairments on quality of life (QOL) measures: BC patients reporting SCC describe a significative impact of cognitive difficulties on perceived quality of life. **p < 0.01; ***p < 0.001.

**Figure 4:**
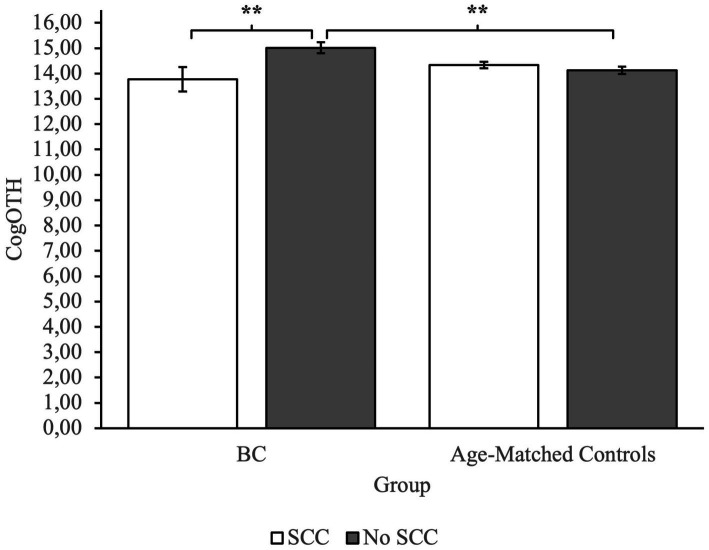
Group comparison between FACTCog comments from others (OTH) measures: BC patients not referring SCC are described by others with more cognitive functionality compared to BC patients referring SCC and age-matched controls not referring SCC. **p < 0.01.

To investigate group differences, we performed an ANOVA. Analysis revealed a main effect of Group on measures of CogPCA and CogQoL, while a main effect of SCC was found only on measures of CogPCA (see Supplementary Table 1 for full results). Most interestingly, we found a highly significant interaction between group membership and SCC on measures of perceived cognitive abilities – CogPCA [*F*(1) = 14.84, *p* < 0.001, *η*^2^ = 0.05], cognitive functionality on quality of life - CogQoL [*F*(1) = 9.90, *p* < 0.01, *η*^2^ = 0.04], and cognitive difficulties referred by others – CogOTH [*F*(1) = 21.95, *p* < 0.01, *η*^2^ = 0.03]. Post-hoc analysis indicated that BC patients referring SCC tended to describe themselves as less cognitive skilled (CogPCA) compared to BC patients not referring SCC (*p* < 0.001), and age-matched controls referring SCC (*p* < 0.001) and not referring SCC (*p* < 0.001; Figure 2). Moreover, BC patients referring SCC report a strong influence of cognitive deficits on quality of life, resulting in a worst perceived ability to cope with everyday activities (CogQoL) compared to BC patients not referring SCC (*p* < 0.01), age-matched controls referring SCC (*p* < 0.001), and age-matched controls not referring SCC (*p* < 0.001; Figure 3). Surprisingly, when comparing comments from others regarding cognitive functionality (CogOTH), post-hoc analysis showed that BC patients not referring SCC are described by others as more cognitive skilled compared to BC patients reporting SCC (*p* < 0.01) and age-matched controls non referring SCC (*p* < 0.01; Figure 4).

### Correlation between cognitive and behavioral, psychosocial, and lifestyle variables in both groups

Tables 3, 4 report the correlations between variables considered in the present study in the two groups separately. PCI20 was eliminated from the correlation table due to its perfect correlation with PCI18 measure [*r*_s_ = 1.00, *p* < 0.001, *N* = 231]. Correlation analysis in the BC group showed a small significant correlation between cognitive reserve - CR and some of the perceived cognitive measures assessed *via* FACT-Cog questionnaire. In detail, the analysis displayed a positive correlation between CR and CogPCA [*r*_s_ = 0.295, *p* < 0.01, *N* = 105], CogOTH [*r*_s_ = 0.204, *p* < 0.05, *N* = 104] and CogQoL [*r*_s_ = 0.214, *p* < 0.05, *N* = 104]. Furthermore, sleep quality – SQI displayed small correlation with CogQoL [*r*_s_ = 0.206, *p* < 0.05, *N* = 105], and a moderate significant correlation with cognitive reserve – CR [*r*_s_ = 0.325, *p* < 0.001, *N* = 105]. Ultimately, Dietary habits – DHI showed a small correlation with cognitive reserve – CR [*r*_s_ = 0.283, *p* < 0.01, *N* = 105], and a moderate correlation with physical activity – PHI [*r*_s_ = 0.430, *p* < 0.001, *N* = 103]. As shown in Table 4, Age-matched control group correlation analysis revealed a small significant correlation between perceived cognitive abilities – PCA and cognitive reserve – CR [*r*_s_ = 0.227, *p* < 0.05, *N* = 226], and dietary habits – DHI [*r*_s_ = 0.180, *p* < 0.05, *N* = 126], while other behavioral and lifestyle factors did not show corelations with any of the cognitive measures investigated. Lastly, physical activity – PHI showed a small significant correlation with dietary habits – DHI [*r*_s_ = 0.210, *p* < 0.05, *N* = 127] and sleep quality – SQI [*r*_s_ = 0.186, *p* < 0.05, *N* = 127].

**Table 3:** Correlation table of cognitive and behavioral, psychosocial, and lifestyle variables in BC patients.

	CogPCI18	CogOTH	CogPCA	CogQOL	CR	DHI	PHI	SQI
CogPCI18	–							
CogOTH	0.447***	–						
CogPCA	0.668***	0.342***	–					
CogQOL	0.508***	0.480***	0.445***	–				
CR	0.172	0.204*	0.295**	0.214*	–			
DHI	0.083	−0.018	0.143	0.094	0.283**	–		
PHI	−0.097	−0.090	−0.042	−0.016	0.074	0.430***	–	
SQI	0.098	0.073	0.189	0.206*	0.325***	0.162	−0.084	–

*p <0.05; **p < 0.01; ***p < 0.001.

**Table 4:** Correlation table of cognitive and behavioral, psychosocial, and lifestyle variables in age-matched controls.

	CogPCI18	CogOTH	CogPCA	CogQOL	CR	DHI	PHI	SQI
CogPCI18	–							
CogOTH	0.583***	–						
CogPCA	0.667***	0.530***	–					
CogQOL	0.360***	0.312***	0.497***	–				
CR	0.054	0.142	0.227*	0.080	–			
DHI	0.123	0.066	0.180*	0.110	0.065	–		
PHI	0.037	−0.106	−0.053	0.080	0.023	0.210*	–	
SQI	0.020	0.022	0.071	−0.016	0.010	0.121	0.186*	–

*p < 0.05; **p < 0.01; ***p < 0.001.

### Levels of cognitive reserve predict subjective cognitive functionality

Results of linear regression analysis conducted in the BC group indicated that cognitive reserve – CR scores significantly predicted performance on perceived cognitive functionality – CogPCA [*R*^2^ = 0.04, *F*(1,102) = 4.43, *p* < 0.05], and comments by others’ regarding cognitive functionality – CogOTH [*R*^2^ = 0.09, *F*(1,103) = 9.83, *p* < 0.01]. A stepwise multiple regression was conducted to evaluate whether measures of cognitive reserve – CR and sleep quality – SQI predict impact of cognition on perceived quality of life – CogQoL. The best model [*R*^2^ = 0.05, *F*(1,102) = 4.88, *p* < 0.05] indicated that only cognitive reserve – CR was a good predictor of impact of cognition on perceived quality of life – CogQoL [*β*^2^ = 0.18, 95% CI (0.01, 0.26), *p* < 0.05].

A further stepwise multiple regression was performed to address the effect of cognitive reserve – CR and dietary habits – DHI on perceived cognitive abilities – CogPCA in the age-matched control group. Analysis revealed that both variables upheld as significant predictors. Two models reached statistical significance. Model 1, including cognitive reserve – CR only [*R*^2^ = 0.04, *F*(1,124) = 6.71, *p* < 0.05], and Model 2, which included both cognitive reserve – CR and dietary habits – DHI [*R*^2^ = 0.08, *F*(1,123) = 4.26, *p* < 0.05]. The addition of a variable significantly improves model prediction, in fact both Cognitive reserve – CR [*β*^2^ = 0.23, 95% CI (0.06, 0.48), *p* < 0.05] and dietary habits – DHI [*β*^2^ = 0.18, 95% CI (0.02, 0.94), *p* < 0.05] found to be significant in the model. In conclusion, Model 2 better predicts perceived cognitive abilities – CogPCA based on psychosocial and lifestyle variables.

## Discussion

The present study aimed to address the prevalence rate of SCC in BC patients using a standardized self-report assessment (FACT-Cog) and to identify psychosocial, behavioral, and lifestyle factors associated with subjective cognitive complaints to determine possible intervention programs aimed at modifiable factors.

### Prevalence rate of subjective cognitive complaints in BC patients

The results indicated a higher prevalence rate of SCC in BC patients compared to a representative sample of women in the same age group without an oncological diagnosis. More than one BC patient out of three (37%) reported a significant decline in subjective cognitive performance after the start of adjuvant therapies, while only one out of six women (17%) without oncological diagnosis reported a decrease in subjective cognitive functionality in the last month. The present results are coherent with previous studies investigating cognitive deficits in BC patients with standardized questionnaires (Schimdt et al., 2016; Whittaker et al., 2022). Analysis revealed that the likeliness of experiencing subjective cognitive deficits in BC patients is nearly three times higher than in an age-matched women sample, thus indicating the importance of SCC assessment in normal care routine for oncological patients. The biological and psychological mechanisms underlying the CRCI phenomena are beyond the nature of the present study, but the results highlight the multifactorial nature of CRCI. The only presence of oncological diagnosis, and the associated therapeutic process, increase the risk of experiencing subjective cognitive deficits. And, albeit subjective cognitive complaints are scarcely correlated to objective cognitive measures (Ahles et al., 2012; Lange et al., 2019), experiencing CRCI in everyday life is strongly associated with lower rates of working life reintegration (Boykoff et al., 2009; Yarker et al., 2010; Duijts et al., 2017), driving capabilities (Myers et al., 2015), and adherence to pharmacological treatments (Zogg et al., 2012; Von Ah et al., 2013; Alatawi et al., 2021), hence making it essential to assess perceived cognitive dysfunctions since the start of oncological diagnostic process to undertake non-pharmacological treatments aimed at modifiable behavioral and lifestyle factors. Moreover, our results on incidence of subjective cognitive impairment on age-matched controls are in agreement with literature investigating role of subjective complaints on general population. Multiple researches (Bassett and Folstein, 1993; Jenkins et al., 2019; Jessen et al., 2020) have highlighted the high heterogeneity of SCC incidence, based on multiple sociodemographic (e.g., age, educational level, etc.) and health-related variables (e.g., psychological issues such as anxiety or depression, or more general health conditions, such as menopause). In conclusion, our results are coherent with studies investigating SCC on adult general population, in particular incidence rate on younger subjects (<65 years old), ranging from 11,2% (Taylor et al., 2018) up to 20% (Mitchell, 2008).

### Influence of SCC on self and others’ perception and impact on the perceived quality of life

Analysis showed that subjects with a BC diagnosis that experience subjective cognitive complaints in everyday life reported lower levels of self-perceived cognitive abilities and that the perceived cognitive deficits had a great role in perceived quality of life. The results suggest that BC patients with SCC tended to describe themselves as less cognitively competent compared to the control group and their lower level of cognitive self-efficacy had a substantial effect on the perceived quality of life. These results are consistent with the literature regarding self-efficacy in BC patients, and its relation to the quality of life (Borjalilu et al., 2017) Self-efficacy plays a crucial role as a mediator of symptom distress (Ziner et al., 2012), and it influences physical and psychological health (Duggleby et al., 2009). Therefore, self-efficacy appears to be a relevant clinical issue in oncology care to prevent mental health problems.

### Differences in behavioral, lifestyle, and psychosocial factors and their role in SCC, self-efficacy, and perceived quality of life

The results demonstrated substantial differences in behavioral and psychosocial factors between BC patients and a group of women without an oncological diagnosis. BC patients had lower cognitive reserve levels compared to controls. Cognitive reserve is defined as a set of social environmental and behavioral factors (e.g., social interactions, leisure activities, education, physical activities) that are protective against cognitive decay and cognitive deficits. An extensive literature (Chapko et al., 2018; Alvares Pereira et al., 2021), suggests a strong correlation between cognitive reserve and preserved cognitive global function in many neurological diseases (e.g., Alzheimer’s disease, Parkinson’s disease, Multiple Sclerosis). However, there is still no agreement regarding the role of the cognitive reserve as a protective factor in cognitive disorders associated with cancer (Romero et al., 2021). Despite the uncertain association between cognitive reserve and CRCI, our study revealed a significant difference in educational attainment between BC patients and age-matched controls. Education is a key factor in cognitive reserve, due to the formation of adaptive thinking and has a positive effect on cognition and subjective cognitive impairment (Chen et al., 2021). The higher percentage of SCC in BC patients could in part derive from different educational levels between the two groups and needs further analysis. Nevertheless, our analysis showed that cognitive reserve plays a crucial role in perceived cognitive deficits. Lower levels of cognitive reserve are associated with lower perceived cognitive abilities, self-efficacy and perceived quality of life in BC patients, indicating that cognitive reserve could have a role as a protective factor in preventing CRCI, and suggesting that specific intervention programs for oncological patients must consider modifiable factors (e.g., leisure, intellectual and social activities) to prevent or delay possible adverse cognitive consequences.

Besides psychosocial factors, our results suggested that the sleep quality of BC patients is worse than in the control group. This finding confirms literature reporting significant sleep disturbances in BC patients (Fortner et al., 2002) and stresses out the higher percentage rate of sleep disorders in the oncological population compared to the non-oncological population, and its relevance in perceived quality of life (Liu et al., 2013).

Surprisingly, no significant differences were found in dietary habits or level of physical activity between BC and the control group. Dealing with cancer could make it harder to maintain an active lifestyle, due to treatment side effects or frequent hospitalizations, thus leading to less physical activity and a less balanced or inappropriate diet. This could be interpreted positively since these two lifestyle factors are already a target of standardized models of aftercare in BC patients.

### Limitations

First, the absence of pre-oncological treatment measures of behavioral, cognitive, and lifestyle measures does not allow to have an objective comparison with a condition without the possible effect of adjuvant therapies. Despite this, given the very short time between the oncological diagnosis and the beginning of adjuvant therapies, even in presence of a pre-treatment evaluation, the strong emotional distress following the diagnosis of oncological pathology would affect the perception of cognitive functionality of patients (Kaiser et al., 2019). Another limitation of the study is due to the absence of measures related to psychological factors such as anxiety, depression, and fatigue. The choice not to include measures of psychological nature was dictated by the desire of the present research to expand the investigation to behavioral and lifestyle factors, given the already extensive literature on the close correlation between psycho-emotional variables and poor perceived cognitive functionality in BC patients (Lange et al., 2019).
Another limitation concerns the observational nature of the present study. Observational studies are typically prone to confounding and selection bias. In fact, due to voluntarily enrollment in the study, our subjects’ sample might be more aware of cognitive functioning and more cofounding variable might have a key role in explaining incidence of CRCI in our sample. But despite selection bias, our results are coherent with incidence of SCC in BC patients (Schimdt et al., 2016; Whittaker et al., 2022), indicating that CRCI is frequently present in breast oncological patients.
  Finally, the recruitment of patients with a specific oncological diagnosis may lead to a poor generalization of the results to other categories of oncological populations. The choice was imputed to the typical course of the disease. Subjects with BC have very high survival rates (87% 5 years after diagnosis), and their life expectancy implies, in most cases, a social reintegration process, with a resumption of pre-diagnosis working and social activities. For this reason, the assessment of cognitive function in this specific type of subject is extremely relevant.

### Implications

Future research should focus on the association between behavioral and psychological factors (e.g., anxiety and depression), to estimate the potential effect of psychological distress and reduced health behavior on quality of life and perceived cognitive functionality. As a more clinical standpoint, current results confirm the multifactorial nature of CRCI and indicate specific behavioral and psychosocial factors for target-oriented intervention to prevent subjective cognitive deficits in BC patients.

## Data availability statement

The raw data supporting the conclusions of this article will be made available by the authors, without undue reservation.

## Ethics statement

The studies involving human participants were reviewed and approved by Ethical Committee of the University of Trieste, Italy n.106 13.07.2020. The patients/participants provided their written informed consent to participate in this study.

## Author contributions

BS, CC, and TW have conceptualized the work. TW, BS, and CC have designed the experimental procedures. RC, SF, and DG selected the patients’ group. TW has performed most of the experiments with data acquisition and wrote the first draft. Interpretation of data were made by TW, BS, and CC. TW prepared the figures and drafted the manuscript which was then revised by BS, CC, and DG, and improved with major and minor suggestions by the other authors. TW performed the statistical evaluation of the data with the supervision of CC and FV. All authors contributed to the article and approved the submitted version.

## Funding

The present study has been funded by Lega Italiana per la Lotta contro i Tumori Programma 5 per mille anno 2019 Progetti di Ricerca in Rete (G75C20000410001).

## Conflict of interest

The authors declare that the research was conducted in the absence of any commercial or financial relationships that could be construed as a potential conflict of interest.

## Publisher’s note

All claims expressed in this article are solely those of the authors and do not necessarily represent those of their affiliated organizations, or those of the publisher, the editors and the reviewers. Any product that may be evaluated in this article, or claim that may be made by its manufacturer, is not guaranteed or endorsed by the publisher.

## Supplementary material

The Supplementary material for this article can be found online at: https://www.frontiersin.org/articles/10.3389/fpsyg.2022.1015573/full#supplementary-material
