# Supplementary material for: Impact of psychosocial, behavioral and lifestyle factors on subjective cognitive complaints and perceived quality of life in a large cohort of Italian breast cancer patients
Source: Front Psychol. 2022 Nov 9;13:1015573. doi: 10.3389/fpsyg.2022.1015573 (PMC9683534; doi:10.3389/fpsyg.2022.1015573)
Supplement: Supplementary file 1 [file Data_Sheet_1.docx]

Supplementary Table 1. ANOVA results

| ANOVA - CogPCA | | | | | | |
| --- | --- | --- | --- | --- | --- | --- |
|  | **Sum of Squares** | **df** | **Mean Square** | **F** | **p** | **η²** |
| Group | 304.82 | 1 | 304.82 | 12.06 | < .001*** | .04 |
| Subjective cognitive complaints | 492.55 | 1 | 492.55 | 19.48 | < .001*** | .07 |
| Group ✻ Subjective cognitive complaints | 357.68 | 1 | 357.68 | 14.15 | < .001*** | .05 |
| Residuals | 5840.13 | 231 | 25.28 |  | | |
| ANOVA – CogQOL | | | | | | |
|  | **Sum of Squares** | **df** | **Mean Square** | **F** | **p** | **η²** |
| Group | 179.75 | 1 | 179.75 | 15.99 | < .001*** | .06 |
| Subjective cognitive complaints | 13.58 | 1 | 13.58 | 1.21 | .273 | .00 |
| Group ✻ Subjective cognitive complaints | 116.78 | 1 | 116.78 | 10.39 | .001** | .04 |
| Residuals | 2574.49 | 229 | 11.24 |  | | |
| ANOVA – CogOTH | | | | | | |
|  | **Sum of Squares** | **df** | **Mean Square** | **F** | **p** | **η²** |
| Group | 1.13 | 1 | 1.13 | 0.39 | .530 | .00 |
| Subjective cognitive complaints | 10.85 | 1 | 10.85 | 3.79 | .053 | .02 |
| Group ✻ Subjective cognitive complaints | 21.66 | 1 | 21.66 | 7.57 | .006** | .03 |
| Residuals | 657.70 | 230 | 2.86 |  |  |  |

Note. * p < .05; ** p < .01; *** p < .001.

Supplementary Table 2. Group comparisons between BC patients based on type of adjuvant therapies: Chemotherapy (alone or + others) vs. Other types of therapies (radiation therapy, hormonal therapy, biological therapy, or combination)

| Independent Samples T-Test | | | | | |
| --- | --- | --- | --- | --- | --- |
|  |  | **Statistic** | **df** | **p** | **Effect Size** |
| CogPCI18 | Mann-Whitney U | 869.50 | -- | .211 | .16 |
| CogPCI20 | Mann-Whitney U | 872.00 | -- | .218 | .16 |
| CogOTH | Mann-Whitney U | 1022.50 | -- | .975 | .00 |
| CogPCA | Student’s t | -2.42 | 104 | .017* | -.55 |
| CogQOL | Mann-Whitney U | 825.00 | -- | .128 | .20 |
| CR | Student’s t | -.16 | 103 | .875 | -.04 |
| DHI | Mann-Whitney U | 834.50 | -- | .126 | .20 |
| PHI | Mann-Whitney U | 831.00 | -- | .187 | .17 |
| SQI | Mann-Whitney U | 790.50 | -- | .065 | .24 |

Note. * p < .05; ** p < .01; *** p < .001.

Supplementary Table 3. Descriptive statistics for BC patients based on type of adjuvant therapies: Chemotherapy (alone or + others) vs. Other types of therapies (radiation therapy, hormonal therapy, biological therapy, or combination)

| Group Descriptives | | | | | | |
| --- | --- | --- | --- | --- | --- | --- |
|  |  |  |  |  |  |  |
|  | **Group** | **N** | **Mean** | **Median** | **SD** | **SE** |
| CogPCI18 | Chemotherapy (alone or + other) | 80 | 57.09 | 61.50 | 12.20 | 1.36 |
|  | Other | 26 | 59.93 | 62.00 | 12.07 | 2.37 |
| CogPCI20 | Chemotherapy (alone or + other) | 80 | 63.54 | 68.50 | 13.70 | 1.53 |
|  | Other | 26 | 66.57 | 69.00 | 13.81 | 2.71 |
| CogOTH | Chemotherapy (alone or + other) | 79 | 14.66 | 15.00 | 1.69 | 0.19 |
|  | Other | 26 | 14.27 | 15.00 | 2.89 | 0.57 |
| CogPCA | Chemotherapy (alone or + other) | 80 | 16.11 | 15.94 | 5.68 | 0.63 |
|  | Other | 26 | 19.21 | 19.06 | 5.57 | 1.09 |
| CogQOL | Chemotherapy (alone or + other) | 79 | 12.50 | 13.00 | 3.60 | 0.40 |
|  | Other | 26 | 13.49 | 14.83 | 3.55 | 0.70 |
| CR | Chemotherapy (alone or + other) | 79 | 20.80 | 21.00 | 5.89 | 0.66 |
|  | Other | 26 | 21.00 | 21.50 | 5.02 | 0.98 |
| DHI | Chemotherapy (alone or + other) | 80 | 6.72 | 7.00 | 1.93 | 0.22 |
|  | Other | 26 | 6.23 | 6.00 | 1.63 | 0.32 |
| PHI | Chemotherapy (alone or + other) | 77 | 2.69 | 2.00 | 1.97 | 0.22 |
|  | Other | 26 | 2.04 | 2.00 | 1.40 | 0.27 |
| SQI | Chemotherapy (alone or + other) | 80 | 3.79 | 4.00 | 2.30 | 0.26 |
|  | Other | 26 | 4.73 | 5.00 | 1.69 | 0.33 |

Supplementary Table 4. Duration of adjuvant therapies in BC patients’ group

| Contingency Tables | | | | | | | |
| --- | --- | --- | --- | --- | --- | --- | --- |
|  | | **Subjective cognitive complaints** | | | |  | |
| **ADJ – Time From Conclusion** | | **No SCC** | | **SCC** | | **Total** | |
| <1yr |  | 8 |  | 4 |  | 12 |  |
| >1yr |  | 19 |  | 9 |  | 28 |  |
| >3yrs |  | 12 |  | 7 |  | 19 |  |
| Ongoing |  | 28 |  | 19 |  | 47 |  |
| Total |  | 67 |  | 39 |  | 106 |  |
| **ADJ - Time From Start** |  |  |  |  |  |  |  |
| <1yr |  | 22 |  | 8 |  | 30 |  |
| >1yr |  | 42 |  | 31 |  | 73 |  |
| >3yrs |  | 3 |  | 0 |  | 3 |  |
| Total |  | 67 |  | 39 |  | 106 |  |
|  | | | | | | | |

Supplementary Figure 1. Scatterplots for significant correlations in BC patients’ group

Supplementary Figure 2. Scatterplots for significant correlations in Age-matched controls’ group
